# Implementing reflective multicriteria decision analysis (MCDA) to assess orphan drugs value in the Catalan Health Service (CatSalut)

**DOI:** 10.1186/s13023-019-1121-6

**Published:** 2019-06-27

**Authors:** Laura Guarga, Xavier Badia, Mercè Obach, Manel Fontanet, Alba Prat, Atonio Vallano, Josep Torrent, Caridad Pontes

**Affiliations:** 1 Àrea del Medicament, Servei Català de la Salut (CatSalut), Travessera de les Corts, 131-159, 08028 Barcelona, Spain; 2 Omakase Consulting S.L., Entença street 332­334 Floor 6, door 4, 08029 Barcelona, Spain; 3 Departament de Farmacologia, de Terapèutica i de Toxicologia, Universitat Autònoma de Barcelona, Av. de Can Domènech, 737, 08193 Cerdanyola del Vallès (Barcelona), Spain

**Keywords:** Multi-criteria decision analysis, Catalan healthcare, Orphan drugs, Decision-making

## Abstract

**Background:**

Orphan medicines show some characteristics that hinder the evaluation of their clinical added value. The often low level of evidence available for orphan drugs, together with a high budget impact and an incremental cost-effectiveness ratio many times higher than drugs used for non-orphan diseases, represent challenges in their appraisal and effective access to clinical use. In order to explore how to handle these hurdles, the Catalan Health Service (CatSalut) began an initiative on a multidimensional assessment of drugs value during the appraisal process. Reflective multicriteria decision analysis (MCDA) using analytical methods was chosen, since it may help to standardise and contextualize all the relevant data related with the drug that could contribute to a decision. The aim of the study was to determine whether the implementation of reflective MCDA methodology could support the decision-making process about orphan medicines in the context of CatSalut.

**Methods:**

The assessment and decision-making process for orphan drugs in the *Programa d’Harmonització Farmacoterapeutica* (PHF) of CatSalut was prioritized to test the implementation of the reflective MCDA both a qualitative and quantitatively. A staged approach was used with the following main steps: selection and structuration of quantitative criteria (Core Model) and qualitative criteria (Contextual Tool), framework scoring and assessment of three orphan drug case studies.

This proof-of-concept would grant a continued refinement of the methodology and, if and when validated, its potential integration to other therapeutic areas of the PHF.

**Results:**

The final framework was composed by 10 quantitative criteria (Core Model) and 4 qualitative criteria (Contextual Tool) according to the PHF goals being the most important criteria “disease severity”, “unmet need”, “comparative effectiveness” and “comparative safety /tolerability”. The matrix developed for the case studies served as a guide for the selection of the essential information that the decision-makers were expected to include in a framework. The reflective discussion was considered the most relevant phase of the approach to support inputs for health decision-making processes reflecting both drug value and place in therapy.

**Conclusions:**

The study showed that reflective MCDA methodology could be implemented to complement the decision-making process in CatSalut, as an aid to determine the clinical added value for orphan medicines. MCDA provided transparency and a structured discussion during the committee meetings, thus increasing transparency and predictability of the relevant items supporting the agreements adopted on orphan drugs access.

## Introduction

In Spain, the decision for price and reimbursement of therapeutic innovations in the basic services portfolio of the public health care system is a national responsibility. However, the other health competences are mainly dependent on the autonomous communities who are responsible for the budget allowance, prioritization and development of measures to ensure efficient access of new drugs considering the characteristics of their own population [1].

In Catalonia, the Catalan Health Care System (*Servei Català de la Salut*, CatSalut) is the regional health care institution responsible for ensuring public access and rights for health delivery. CatSalut runs a specific program for drug evaluation and decision-making, *Programa d’Harmonització Farmacoterapeutica* (PHF) aimed to prioritize innovative drugs according to their added value and considering the needs and budgeting priorities, while to guaranteeing equity in the access to treatments throughout Catalonia. In addition to establish the relative effectiveness, PHF also specifies the clinical criteria recommendations and the place in therapy of the new drug in relation to available therapies [2]. A dedicated committee composed by 18 members (6 physicians, 6 healthcare service managers, 5 pharmacists and 1 patient representative) was specifically devoted to decision-making regarding of Orphan Drugs at the time of the present exercise.

To address the PHF goals, CatSalut began an initiative in 2015 focused on a multidimensional assessment of drugs value [3]. Among all the multicriteria approaches, the reflective multicriteria decision analysis (MCDA) was chosen since, through analytical methods, helps to contextualize all the relevant data related with the product and which could contribute to a decision [4]. Reflective MCDA framework (EVIDEM) is composed by a set of criteria derived from the ethical imperatives of healthcare and their relative importance under a transparent and standardised process which promote the reflection of the stakeholders and facilitate the sharing of diverse perspectives [5, 6].

The first step was focused on adapting and assessing the value of a decision reflective MCDA framework for orphan drugs evaluation from CatSalut perspective. The standard evaluation and decision-making procedure of CatSalut was compared with the reflective MCDA methodology and was adapted to the local context. Finally, it was concluded that reflective MCDA could be a useful and feasible tool to complement the current evaluation methods of CatSalut, contributing to standardisation and pragmatism, providing a method to tackle ethical dilemmas and facilitating discussions related to decision-making [7]. This initiative was included in the Health Plan of Catalonia for 2016–2020 [2].

Thus, the aim of the present study was to assess the feasibility of the implementation of a reflective MCDA methodology to support orphan drug decision-making process to the Catalan context.

## Methods

A qualitative study was carried out with a staged approach. The following main steps were used: 1) selection and structuration of quantitative criteria (MCDA core Model) and qualitative criteria (Contextual Tool), 2) weighting of the quantitative criteria of the framework and 3) testing through assessment of three orphan drug case studies.

### Selection and structuration of criteria for orphan drugs framework

An updated specific MCDA framework for orphan medicines evaluation was developed considering the suggested quantitative and qualitative criteria of the pilot framework previously developed (December 2015), [7] and additional criteria identified through the following sources:

- Updated version of EVIDEM framework (v.4): New considerations of the EVIDEM framework which is continually updated by the EVIDEM collaboration group to support pragmatic and reflective healthcare decision-making [8]
- Systematic Literature Review (SLR): Criteria from a SLR conducted by the *Instituto Carlos III* (ISCIII). The study identifies and analyses the information about the reimbursement criteria and pharmaceutical policies used for orphan drugs decision-making applied in the main countries of the European Union, Australia and Canada. Their search strategy covered literature published between to 2004 and 2015 [3].

The abovementioned resources were analysed in order to assess the feasibility of their adaptation for the inclusion in the current PHF. The analysis was performed by two of the authors (XB and LG).

The final version of the framework was discussed and validated indicating whether a criterion should or should not be systematically considered when appraising a orphan drug in the setting of the PHF by 5 members of PHF (physicians, pharmacists and health service managers). Definition of criteria is similar to EVIDEM framework focused on orphan drugs [8].

### Weighting of orphan drugs framework (value system elicitation)

The weighting of the quantitative criteria of the final orphan drugs framework was done by a subgroup of representatives of the committee of CatSalut in charge of decision-making processes (clinicians and hospital pharmacist during a workshop performed on July 2017 in Barcelona). This subgroup was selected to enable the properly field testing of the orphan drug framework and was trained on reflective MCDA methodology previously to participate in the panel.

A direct rating scale (five-point weighting technique) was used where each participant gave a relative weight per criterion using a nonhierarchical simple 5-point scale (1 = lowest relative importance, 5 = highest relative importance) [8, 9].

#### Data analysis

All data analysis was carried out in Microsoft Excel. Criteria weights were normalized to sum up to 1 for each participant: for the 5-point rating scale method, each weight was divided by the sum of weights across all criteria; for the point allocation method, criteria ratings were multiplied by domain weight and rescaled to range from 0 to 1 [8].

### Case studies – appraisal of three orphan drugs

Three orphan drugs approved in Europe were chosen to test the suitability of the proposed MCDA framework to capture all the relevant dimensions and criteria used for discussion. The medicines selected were chosen from medicines already scheduled for assessment by the PHF, and considering the fact that the 3 drugs differed in terms of size of target population, availability of treatment alternatives, quantity and quality of the supporting evidence and economical impact, among others, The available evidence was summarized for each orphan drug in the evidence matrix, following the reflective MCDA methodology [8].

During the workshop performed on July 2017, the participating PHF members rated individually the three orphan drugs matrix for each drug assessment, considering the Catalan healthcare context.

#### Data analysis

Data were collected individually, transferred to a common database and analysed with Microsoft Excel. A descriptive analysis of the value of each criterion was conducted separately. Non-comparative criteria were rated in a 0 (worst) to 5 (best) scale and comparative criteria (efficacy, safety, PRO (Patient Reported Outcomes) and cost) was rated in a scale from − 5 to 5. For each criterion, the mean, standard deviation (SD) and range of minimum and maximum scores were calculated. The value contribution (V_x_) of each quantitative criterion was then calculated as the product of its normalised scoring (W_x_, ∑ W_x_ = 1) and standardised score (non comparative criteria: S_x_ = score/5 or comparative criteria: S_x_ = score/10). The overall MCDA value estimate (V) of each orphan drugs was calculated based on a linear additive model as a sum the value contributions (V_x_) of all (n) criteria of the quantitative criteria [8, 10]:

$$ \mathrm{VE}=\sum \limits_{x=1}^n{\mathrm{VC}}_x=\sum \limits_{x=1}^n\left({\mathrm{W}}_x\times {\mathrm{S}}_x\right) $$

## Results

### Selection and structuration of the criteria for orphan drugs framework

The pilot framework developed in the previous study [7] considers a set of criteria structured into two distinct sections: MCDA Core Model, composed of 13 quantitative criteria selected for the assessment of the drug and a MCDA Contextual tool, composed of 4 qualitative criteria that consider the context surrounding decision-making (Table 1).

**Table 1:** Criteria considered in the pilot framework

Criteria	Type of criteria	Source
Disease severity	Quantitative	Previous study [7]
Size of affected population	Quantitative	Previous study [7]
Unmet needs	Quantitative	Previous study [7]
Comparative effectiveness	Quantitative	Previous study [7]
Comparative safety/tolerability	Quantitative	Previous study [7]
Comparative patients perceived health (PRO)	Quantitative	Previous study [7]
Type of preventive benefit	Quantitative	Previous study [7]
Type of therapeutic benefit	Quantitative	Previous study [7]
Cost of orphan drug	Quantitative	Previous study [7]
Other medical costs	Quantitative	Previous study [7]
Non-medical costs	Quantitative	Previous study [7]
Quality of evidence	Quantitative	Previous study [7]
Expert consensus / clinical practice guidelines	Quantitative	Previous study [7]
Population priorities and access	Qualitative	Previous study [7]
Common goal and specific interests	Qualitative	Previous study [7]
Opportunity costs and affordability	Qualitative	Previous study [7]
System capacity and appropriate use of intervention	Qualitative	Previous study [7]

*PRO* Patient Reported Outcomes

In addition, seven new criteria were analysed by the PHF members in order to decide their introduction or modification in the updated version of the framework (Table 2).

**Table 2:** Additional criteria considered for orphan drugs framework

Criteria	Type of criteria (quantitative/qualitative)	Source
Size of affected population	Quantitative	Previous study [7]
Preventive benefit	Quantitative	Previous study [7]
Therapeutic benefit	Quantitative	Previous study [7]
Non-medical costs	Quantitative	Previous study [7]
Budget Impact	Quantitative or qualitative	EVIDEM framework v.4 [8] and SLR [3]
Rarity	Quantitative or qualitative	SLR [3]
The rule of rescue	Quantitative or qualitative	SLR [3]

*SLR* Systematic Literature Review

All the criteria from Table 2 were accounted to assess if they could complement the orphan drugs framework. Some considerations were provided by PHF members regarding the goals and content of the program:

- Size of affected population: The ethical basis of this criterion reflects an aspect of the utilitarianism principle (greatest good for greatest number). Orphan drugs target small populations. Then, this principle, albeit important, need to be considered as a low priority, in order to minimise differences between rare and ultra-rare diseases. It was agreed to exclude the criteria.
- Preventive benefit: the vast majority of rare diseases are of genetic origin and interventions are therefore symptomatic or curative rather than preventive. It was concerted to exclude the criteria.
- Therapeutic benefit: Based on its definition, the nature of the clinical benefit provided for patient-level intervention (i.e, symptom relief, prolongation of the life, healing), the criterion was maintained in the framework. However, it will be redefined so that it captures the therapeutic benefit in a broad and far-reaching way, including aspects such as reduction of risk factors, reduction in disease transmission, etc.
- Non-medical costs: Although non-medical expenses are highly relevant to rare diseases, the lack of information and the difficulty to quantify them makes them difficult to consider. It was agreed to exclude the criteria.
- Budget Impact: Relevant criteria for drug assessment in PHF process. It was agreed to include the criteria to the domain “feasibility contextual criteria” (Contextual tool). Furthermore, its inclusion supports the exclusion of the criteria “size of the population”, which would be covered with the financial/budgeting exercise.
- Rarity: The concept of common disease, rare or ultra -rare, is closely related with the number of patients affected. To be consistent with the exclusion of “size of affected population” criteria it was agreed not to include this criterion.
- The rule of rescue: The criterion describes the perceived duty to save endangered life whenever possible although the drug is not indicated or cost-effective. It was agreed to exclude the criteria.

The final orphan drugs framework validated by participants is shown as Table 3.

**Table 3:** MCDA Core Model (criteria appraised quantitatively) and MCDA Contextual Tool (criteria appraised qualitatively)

MCDA Core Model	
Domains	Criteria
Disease Impact	Disease severity
	Unmet needs
Comparative outcomes of orphan drugs	Improvement of efficacy/effectiveness
	Improvement of safety/ tolerability
	Improvement of patient perceived health/PRO
	Type of therapeutic benefit
Economic consequences of intervention	Annual patient cost of treatment
	Other medical costs
Knowledge about intervention	Quality of evidence
	Expert consensus/clinical practice guidelines
MCDA Contextual Tool	
Domains	Criteria
Normative contextual criteria	Population priorities and access (principle of equity)
	Common goal and specific interests
Feasibility contextual criteria	System capacity and appropriate use of orphan drugs
	Opportunity costs and affordability (budget impact)

*MCDA* Multiple Criteria Decision Analysis

### Weighting of orphan drugs framework (value system elicitation)

**Fig. 1:**
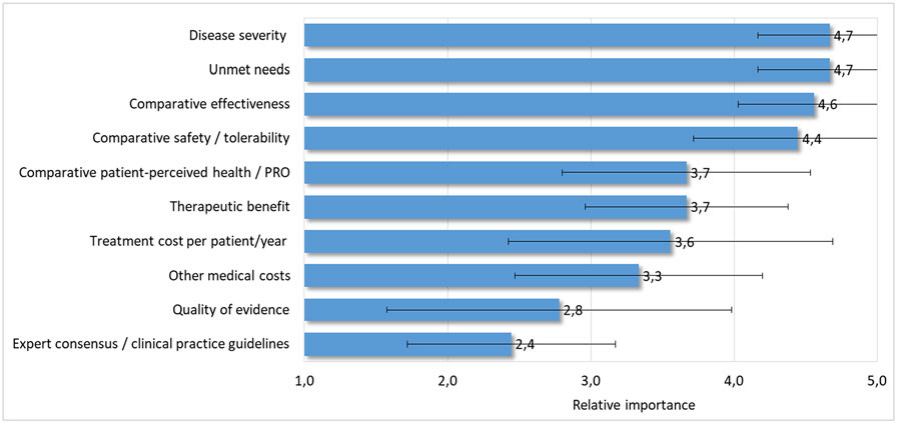
Mean (SD) of level of importance rated by participants to final orphan drugs framework. SD: standard deviation; PRO: Patient Reported Outcomes

According to the results collected from the 5-point weighting scale (Fig. 1), the more important criteria for orphan drugs were “disease severity and unmet needs” (4,7 points ± 0,5) followed by “comparative effectiveness” (4,6 points ± 0,5) and “comparative safety/ tolerability” (4,4 points ± 0,7). The least important criteria were “expert consensus /clinical practise guidelines” (2,4 points ± 0,7) and “quality of evidence” (2,8 points ± 1,2).

### Case studies – appraisal of 3 orphan drugs

The next step involved in the implementation approach of the framework in the drug review process was appraising three orphan drugs: alpha 1-antitrypsin for alpha1-antitrypsin deficiency, eliglustat for Gaucher disease and tolvaptan for autosomal dominant polycystic kidney disease.

The matrix developed for the case studies served as a guide for the relevant information that decision-makers expect to include in a framework.

**Fig. 2:**
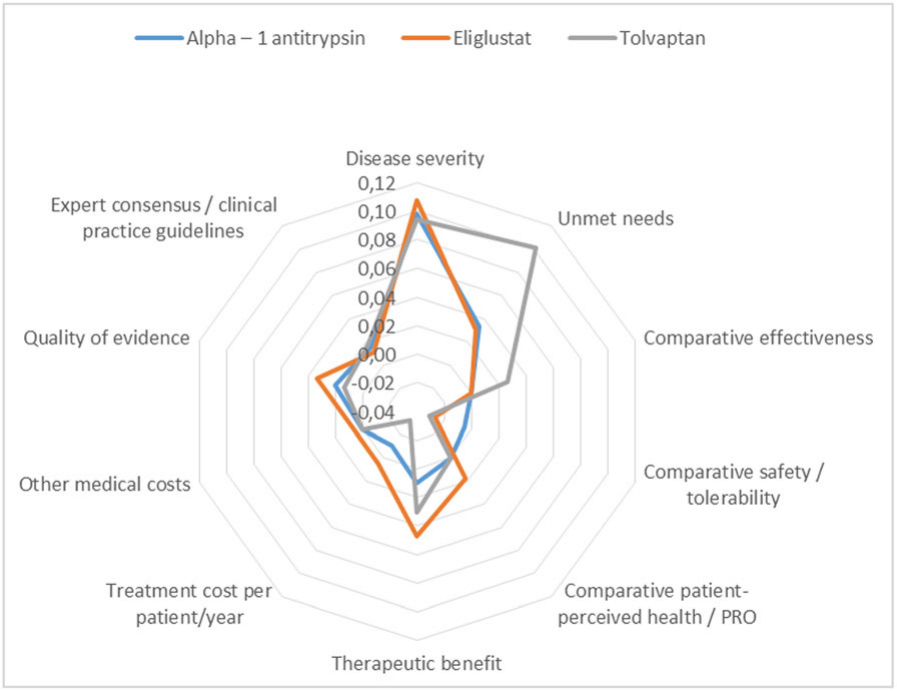
Comparison of value contribution of each criterion: alpha – 1 antitrypsin, tolvaptan and eliglustat. PRO: Patient Reported Outcomes

**Fig. 3:**
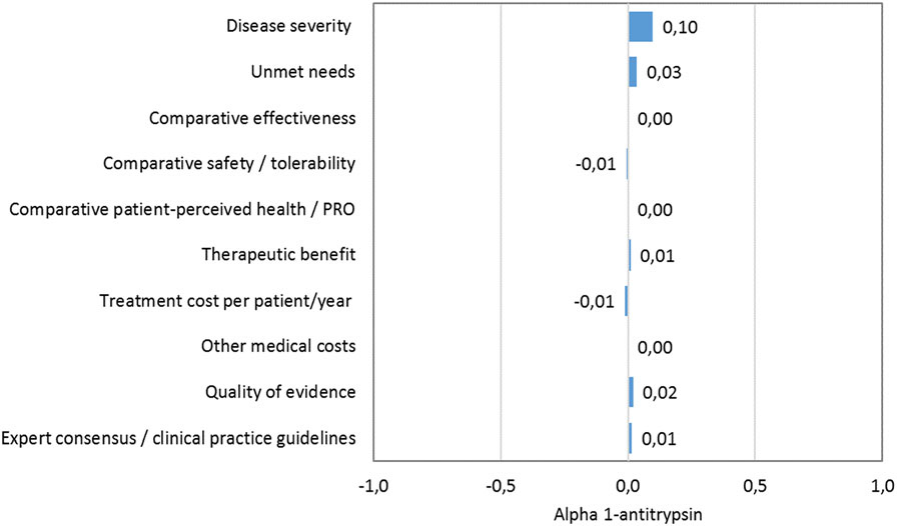
Value of contribution of each criteria: alpha – 1 antitrypsin vs BSC. PRO: Patient Reported Outcomes

**Fig. 4:**
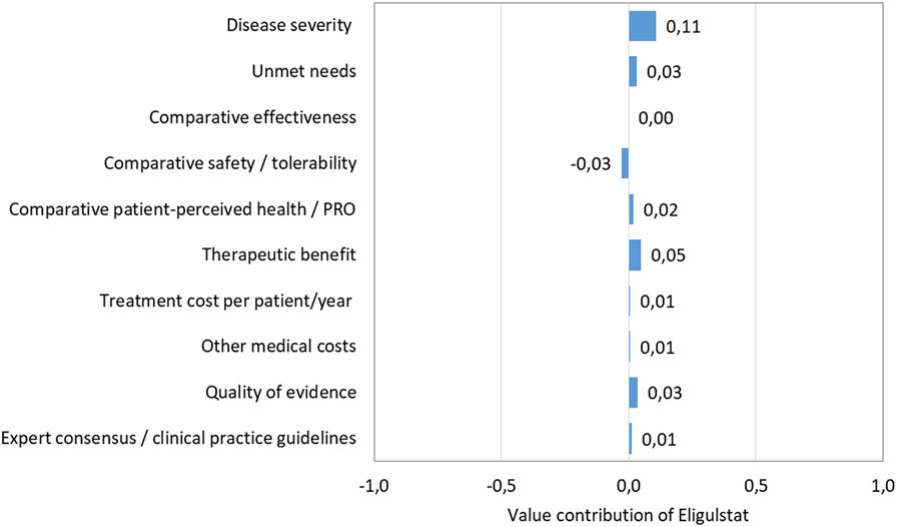
Value of contribution of each criteria: eliglustat vs imiglucerase. PRO: Patient Reported Outcomes

**Fig. 5:**
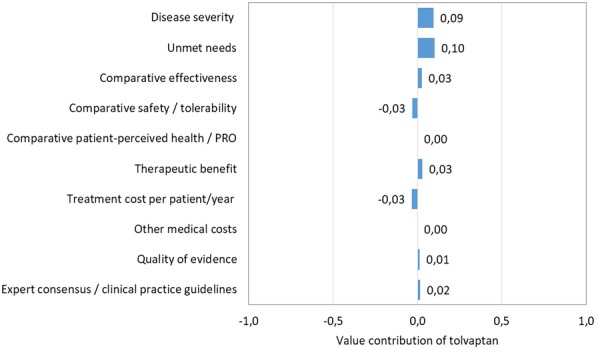
Value of contribution of each criteria: tolvaptan vs placebo. PRO: Patient Reported Outcomes

The overall scores achieved for each orphan drug, including the contribution of the individual criteria scores to the total are depicted in Figs. 2, 3, 4 and 5.

- Disease severity of the *alpha – 1 antitrypsin deficiency* was considered high, reflecting the perception of their impact on mortality and morbidity. Alpha – 1 antitrypsin was not considered to have an added value versus standard of care in regards to efficacy, safety and quality of life (QoL). Regarding the type of benefit, alpha – 1 antitrypsin was considered as not providing an added therapeutic benefit, considering improvements on clinical variables or imaging abnormalities. Comparative treatment cost and other medical costs were assessed with a neutral score. Related to its inclusion in the international guidelines and quality of evidence for alpha – 1 antitrypsin was considered moderate, because of uncertainty in the methodology and results of the clinical trial publication.
- *Gaucher disease* was considered a very severe disease reflecting a high estimated mortality and co-morbidities because of impairment of relevant organs such as liver, spleen, bone marrow and bones. Some enzyme replacement therapies have been approved for Gaucher disease: imiglucerase, velaglucerase and miglustat. Eliglustat is an oral treatment that was considered similar to the previously approved drugs in regards to the therapeutic efficacy, but with the advantage of oral route despite a worst safety profile, although possibly better than the other oral alternative miglustat. The QoL outcomes were seen as translating this fact in a benefit for the patients. Treatment cost per patient and other medical costs were assessed with slightly positive score to eliglustat. Quality of evidence was considered low. Eliglustat was not included in the spansish recommendation about Gaucher disease [11] management since by the time of the guideline publication eliglustat was not yet approved by the European Medicines Agency (EMA).
- *Autosomal dominant polycystic kidney disease* was perceived as a very severe disease with an unfavourable prognostic based on reduced survival of these patients, and the lack of effective alternatives. It was observed that the efficacy of tolvaptan was moderately better than placebo although it is accompanied with a worst safety profile. There was no published data related to QoL, for this reason the score was considered neutral. Regarding the type of benefit tolvaptan was judged to provide a moderate therapeutic benefit. Comparative treatment cost was assessed with a negative score to tolvaptan and other medical costs with neutral score given the lack of available evidence related to these criteria. Quality of evidence was considered very low due to the poor external validity or applicability. The ERA-EDTA (European Renal Association and European Dialysis and Transplant Association) recommended tolvaptan with some clinical criteria of use.

The overall MCDA value estimates (V) of each orphan drug were 0.16, 0.23 and 0.22, for alpha-1 antitrypsin, eliglustat and tolvaptan, respectively. The utility of the overall MCDA value estimates was discussed, and the PHF members considered that MCDA contributed most to decision making process when criteria were described separately, rather than described through a summary score. The PHF members considered that a structured approach to multiple criteria allowed focusing discussion into most relevant domains and criteria, avoided potential linking between numeric thresholds and decisions, and thus added most value from the multidimensional approach to their process of decision-making.

## Discussion

The outcome of this study provides valuable insight on the implementation of the multicriteria orphan drug assessment in the CatSalut context. MCDA would be feasible to support the assessment of reflective value of new orphan drugs, and may be useful to structure and conduct a deliberate the discussion during the PHF committee meetings.

Evaluation and decision-making for orphan drugs is a challenge, due to the inherent difficulties to generate a high-quality evidence of value in small populations. For this reason, the management of orphan drugs often implies areas of substantial uncertainty. Such scenario was suitable for testing whether the reflective multicriteria tool might be able to provide perspective and facilitating decision-making through a systematic, consistent and integrated approach to several dimensions of assessment. MCDA allows visualizing the criteria that may be not explicitly informed during the decisions reached in medicines evaluation committees meetings, such as unmet needs, severity of the disease and QoL, as well as other contextual variables which could contribute to reach a final decision considering the complexity of the drug, the potentially treated population and/or the complexity of its environment.

Reflective MCDA can be used to quantify the importance of the different criteria of assessment and the relevance assigned to specific items. Furthermore, it was regarded that quantitative modelling had to be supported by a qualitative descriptive approach, similar to a process of consensus. Reflective MCDA allowed to identify the range of results for the dimensions and for each assessment criteria, as well as the relevance assigned to specific results. Theses ranges and results were used to prepare qualitative discussion in a structured qualitative approach that is designed to support the identification and communication of the most relevant criteria.

MCDA in this case would not be used as a formula to come to a decision, but rather as a tool to complement the values and viewpoints of committee members, allowing visualization and traceability of key decision elements and making them transparent and supportive of the reflection during discussion across the range of dimensions in the overall assessment of a new drug, ensuring a systematic and thorough procedure. The approach helps to focus the discussion, providing a common structure which is similar across products. Also allows to identify points with diverging opinions from the multidisciplinary decision-making committee.

Another strength of MCDA is that it is suited to incorporate the interpretation of the evidence from different perspectives (reasoning the scoring and weights value), and the methodology has the advantage of providing sufficient flexibility to incorporate relevant items tailored to singular cases and/or situations that may be incorporated rapidly to PHF process within the established framework. Although the MCDA was adapted to the process of the PHF, the adaption is tailored to our process, and may be non-generalizable to other purposes or contexts. However, similar processes can be made to adapt MCDA in other settings.

There have been attempts in Europe to implement reflective MCDA approaches to support managed deliberation through considerations, avoiding the way predominant group members can take over decision-making [12].

Friedmann et al. [12], reviewed the existing evidence regarding the use of MCDA in the assessment of orphan drugs worldwide. A total of seven articles suggest that MCDA is increasingly being used in the context orphan drug assessment. MCDA demonstrate to be a flexible assessment with the potential to assisting decision-making regarding reimbursement for orphan drugs [12]. The main findings of the 7 articles reviewed by Friedmann et al. report the use of the EVIDEM framework in several studies, with criteria concerning disease-specific, health intervention-specific and contextual factors. In our study “disease severity” and “clinical efficacy/effectiveness” were considered the most relevant criteria for the assessment of orphan drugs, similarly than reported by other institutions [12]. Additionally, our results considered the criterion “unmet clinical need” as very relevant. In contrast, in previously published studies “Other comparative costs” and “treatment innovation” were the criteria considered as less important [12], while in our exercise the less relevant criteria were “quality of evidence” and “expert consensus/clinical practise guidelines”.

In Italy, the Lombardy health directorate has used a reflective multicriteria approach combined with the European network for health technology assessment (EUnetHTA) core model to appraise health technologies and make health funding coverage decisions since 2012. The interaction with stakeholders was facilitated by a transparent process and demonstration of reasonableness. The process was evolving to further increase legitimacy by involving a wider array of stakeholders such as citizens or patients [4, 13].

The implementation of a reflective MCDA methodology to support orphan drug decision-making follows one of the nine recommendations set up by the European Working Group for Value Assessment and Fundings Processes in Rare Diseases (ORPH-VAL), which propose that orphan drug assessment should consider all relevant elements of product value in an appropriate multi-dimensional framework [14].

The current study has some limitations. The constitution of only a subgroup representative of committee to enable the properly test of the tool could imply that not all the different perspectives were taken into account during the discussion (health economists, bioethical and patient representatives didn’t participate in the study).

Another limitation of the study was that the scenario adopted just focused on orphan drug assessment, which for their particular challenges, led to exclusion of some criteria relevant for common diseases. The scope of CatSalut committee is to take decisions on both rare and common diseases showing that the framework needs to be expanded from orphan drugs to non-orphan drugs medicine products.

Additionally, another limitation was the description of the concept and definitions of each criterion which could have been more explicit to reduce the variability of the interpretation by the decision-makers. Such variability might have conditioned different results for the scoring and weighting phases.

Finally, it is important to highlight that the synthesis of the evidence to develop the evidence matrix could minimise the advantages of the tool, which are focused on the identification of the variety of points of views and promotion of a multidimensional reflective discussion.

## Conclusion

This study shows that MCDA methodology could become a systematic procedure to complement the overall process of assessment of the added clinical value of innovative medicines by CatSalut.

There is a growing understanding that the assessment based only on traditional criteria such as clinical efficacy, safety and cost, does not allow the value of a new drug to be fully captured. Considering the goal to promote the quality of drug prescription, MCDA allows a deep exploration of a set of quantitative and qualitative inputs, including reflective discussions and individual comments facilitating the evaluation of innovative drug, positioning medicines within therapeutic algorithms and allowing equitable resource allocation in the healthcare sector.

By integration of MCDA to the PHF procedures, MCDA could support the technical evaluation report providing a structured, standardised and transparent approach, contextualising the relevant data for each drug and facilitating evidence and ethical-based discussions among all the different stakeholders involved in evaluation and decision-making purposes.

## Data Availability

Not applicable.

## Abbreviations

Catsalut: Catalan Health Service *(Servei Català de la Salut)*
EMA: European Medicines Agency
ERA-EDTA: European Renal Association and European Dialysis and Transplant Association
EUnetHTA: European network for health technology assessment
MCDA: Multiple Criteria Decision Analysis
ORPH-VAL: European Working Group for Value Assessment and Fundings Processes in Rare Diseases
PHF: Pharmacotherapeutic Harmonization Program (*Programa d’Harmonització Farmacoterapeutica)*
PRO: Patient Reported Outcome
QoL: Quality of life
SD: Standard deviation
SLR: Systematic Literature Review
